# Neogene origins and implied warmth tolerance of Amazon tree species

**DOI:** 10.1002/ece3.441

**Published:** 2013-01-10

**Authors:** Christopher W Dick, Simon L Lewis, Mark Maslin, Eldredge Bermingham

**Affiliations:** 1 Department of Ecology and Evolutionary Biology, University of Michigan830 North University Avenue, Ann Arbor, MI, 48109-1048; 2 Smithsonian Tropical Research InstituteP.O. Box 0843-03092, Balboa Ancón, Republic of Panamá; 3 Earth and Biosphere Institute, School of Geography, University of LeedsLeeds, LS2 9JT, U.K; 4 Department of GeographyPearson Building, Gower Street, UCL, London, WC1E 6BT, U.K

**Keywords:** Amazon forests, comparative phylogeography, ecological niche models, global change, molecular clock, thermal tolerance, tropical trees

## Abstract

Tropical rain forest has been a persistent feature in South America for at least 55 million years. The future of the contemporary Amazon forest is uncertain, however, as the region is entering conditions with no past analogue, combining rapidly increasing air temperatures, high atmospheric carbon dioxide concentrations, possible extreme droughts, and extensive removal and modification by humans. Given the long-term Cenozoic cooling trend, it is unknown whether Amazon forests can tolerate air temperature increases, with suggestions that lowland forests lack warm-adapted taxa, leading to inevitable species losses. In response to this uncertainty, we posit a simple hypothesis: the older the age of a species prior to the Pleistocene, the warmer the climate it has previously survived, with Pliocene (2.6–5 Ma) and late-Miocene (8–10 Ma) air temperature across Amazonia being similar to 2100 temperature projections under low and high carbon emission scenarios, respectively. Using comparative phylogeographic analyses, we show that 9 of 12 widespread Amazon tree species have Pliocene or earlier lineages (>2.6 Ma), with seven dating from the Miocene (>5.6 Ma) and three >8 Ma. The remarkably old age of these species suggest that Amazon forests passed through warmth similar to 2100 levels and that, in the absence of other major environmental changes, near-term high temperature-induced mass species extinction is unlikely.

## Introduction

Warmth from anthropogenic greenhouse gas emissions may severely impact Amazon trees because the latitudinal distance species would need to move to maintain a constant temperature is large, implying that many species may be unable to track the future climate and become extinct (Colwell et al. 2008; Jones et al. 2009). More formally, climate envelope modeling arrives at a similar conclusion, but because upper-bound heat-tolerance in these models cannot exceed the observable maximum current surface air temperature, predicted extinctions may be inflated (Feeley and Silman 2010; Corlett 2011). Moreover, experiments show that tropical plants can photosynthesize and maintain a positive carbon balance under higher temperatures than those occurring today (Krause et al. 2010; Way and Oren 2010), thus suggesting that persistence may be possible for many lowland rain forest tree species. A complementary approach to assist in distinguishing the likely thermal tolerance of Amazon tree species is to consider historical evidence, because at times in the past, Amazon surface air temperatures have been higher than those today (Feeley and Silman 2010; Hoorn et al. 2010; Jaramillo et al. 2010; Haywood et al. 2011).

Some 56.3 Ma during the Paleocene-Eocene Thermal Maximum (PETM), global mean temperature increased by 5–6°C over a period of ≤20 ka (Haywood et al. 2011). Although many marine species became extinct, fossil pollen from the PETM showed an increase in tree diversity in three South American rainforest sites with abundant rainfall (Jaramillo et al. 2010). The mid-Miocene climatic optimum (13–15 Ma) was the warmest time of the Neogene (the ca. 20 Ma Period encompassing the complete Pliocene and Miocene Epochs; (Zachos et al. 2001). More recently, the early Pliocene (3.6–5.3 Ma) and parts of the Late Miocene (8–10 Ma) had surface air temperatures similar to IPCC predictions for the year 2100 under low and high CO_2_ emissions scenarios, respectively (IPCC 2007; You et al. 2009; Haywood et al. 2011). Fossil evidence suggests that megathermal (frost intolerant) forests were globally more extensive during the Neogene (Morley 2000) and possibly harbored higher levels of tree diversity than today's tropical forests (Hoorn et al. 2010). However, the relevance of Neogene warmth to the thermal tolerance of modern tropical trees depends, in part, on establishing the age of the tree species and their prior geographic distributions. If contemporary species extend back to the Pliocene or Late Miocene and did not have access to upper elevation thermal refugia, then they have likely survived air temperatures as high as or higher than those predicted for the coming decades.

Here, we use a phylogeographic approach to determine if 12 contemporary Amazon tree species have endured historical air temperatures that exceed current thermal maxima. Because many of the 12 study species are rain forest specialists, their history provides proxy information about tropical rain forest distributions through time.

## Materials and Methods

### Study species and collection sites

**Figure 1:**
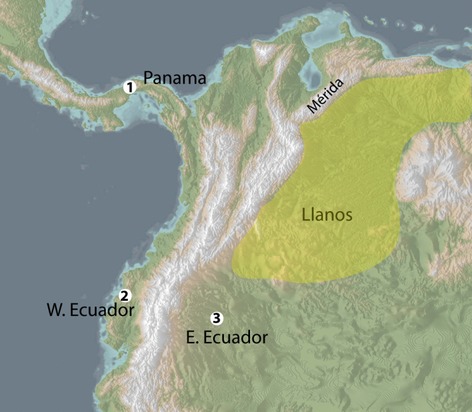
Primary collection sites in (1) central Panama, (2) Western Ecuador (Esmeraldas Province), and (3) Amazonian Ecuador (Yasuní National Park). Additional collections were made in Brazil, Peru, French Guiana, and Bolivia for some species. The Andes and the llanos region presently form a strong geographic barrier between lowland moist forests, east and west of the Andes. The uplift of the Merida cordillera occurred roughly at the Pliocene–Pleistocene boundary (ca. 2.7 Ma).

We obtained DNA sequences from the ITS and cpDNA genes and spacer regions from geographic samples of 12 species (12 genera; 8 families) from the Amazon basin, Chocó region and Central America (Table 1; Fig. 1). The sampling sites were located on either side of the northern Andes Mountains, which have been a geographic barrier between Chocó and Central America (trans-Andes) and the Amazon basin (cis-Andes) through the Neogene (Hoorn et al. 2010), but more significantly so following the Merida uplift of the eastern cordilleras at the Pliocene/Pleistocene boundary, ca. 2.7 Ma (Helmens and van der Hammen 1994), which blocked a potential lowland dispersal route along the Caribbean coast (Brumfield and Capparella 1996) (Fig. 1).

**Table 1:** Characteristics of study species, including geographic range (N = Neotropics, AA = Amphi-Atlantic (Africa + Neotropics), C = Caribbean Islands, At = Brazilian Atlantic Forests), successional stage (P = pioneer, GS = gap specialist; T = shade tolerant), and stature (C = canopy, U = understory, CE = canopy emergent) (*drought tolerant species), mean and standard error of tMRCA estimate, and 95% highest posterior density interval (HPD) (Bayesian analog of confidence intervals)

Latin binomial	Geographic Range	Stage, Stature	tMRCA (±SE) (Ma)	Upper, Lower 95% HPD (Ma)
*Ceiba pentandra*	AA, C	P, CE*	0.2223 (0.00585)	0.00014, 0.6975
*Andira inermis*	AA, C	GS, C*	1.4055 (0.00788)	0.3713, 2.7062
*Trema micrantha*	N, C	P, U*	1.9167 (0.00905)	0.53, 3.529
*Brosimum guianense*	N, At	T, C	2.9172 (0.01067)	1.2462, 4.8233
*Palicourea guianensis*	N, C, At	GS, U*	3.0295 (0.01268)	1.1007, 5.2625
*Cupania cinerea*	N	T, C*	6.4363 (0.01778	3.3458, 9.5587
*Garcinia madruno*	N	T, C	6.9458 (0.01492)	4.3041, 9.6291
*Celtis schippii*	N	T C	6.9848 (0.01663)	4.0696, 10.0757
*Symphonia globulifera*	AA, C, At	T, C	7.0882 (0.01773)	4.3201, 10.3423
*Ochroma pyramidale*	N, C	P, C*	8.0611 (0.01588)	5.1609, 10.9799
*Poulsenia armata*	N	T, C	8.9379 (0.01882)	5.58, 12.4139
*Chrysophyllum argenteum*	N, C	T, C	9.8766 (0.02049)	6.4041, 13.9039

Over 60% of the tree species (433 species) in Central Panama exhibit a cross-Andean disjunction and harbor Amazon basin populations (Dick et al. 2005). The 12 study species were selected by comparing tree species lists from Center for Tropical Forest Sciences (CTFS) forest inventory plots in Barro Colorado Island, Panama (BCI) and Yasuní National Park, Ecuador (YAS). Trees were collected outside of the plots in both sites, and from remnant forests on the Pacific coast of Ecuador (Esmeraldas Province, ESM). Phylogeographic analyses were performed on >5 trees per species per site. Additional collections were obtained from herbarium specimens representing accessions from Costa Rica, Peru, Bolivia, Brazil, and French Guiana (Table S1). Voucher collections from ESM are stored in Quito (QCNE); Vouchers from YAS and BCI are stored at PUCE, MICH, and PAN herbaria.

Species taxonomic boundaries have been delimited in recent monographs. The species have broad geographic ranges within the Neotropics, with a subset of species occurring in the West Indies and/or Africa. *Andira inermis* (W. Wright) H.B.K. (Fabaceae: Papilionoideae) is a canopy tree (height 10–15 m) of lowland forests in Africa and the Neotropics. *Brosimum guianense* (Aubl.) Huber (Moraceae) is a canopy tree (10–40 m) of mature *terra firme* and seasonally flooded forests. *Ceiba pentandra* (L.) Gaertn. (Malvaceae), kapok tree, is a wind-dispersed and bat-pollinated canopy emergent tree (≥60 m) of dry and moist forests of the Neotropics and Africa (Dick et al. 2007). *Celtis schippii* Standl. (Cannabaceae) is a canopy tree (≤30 m) of mature lowland Neotropical forest. *Chrysophyllum argenteum* Jacq. (Sapotaceae) is a canopy tree (20–25 m) of lowland to premontane wet forests. *Cupania cinerea* Poepp. (Sapindaceae) is a mid-canopy tree (8–10 m) of upland and flooded forests in the continental Neotropics and West Indies. *Garcinia madruno* (Kunth) Hammel (Clusiaceae) is a sub canopy tree (5–20 m) of mature moist forests. *Ochroma pyramidale* (Cav. ex Lam.) Urb. (Malvaceae), balsa, is a wind-dispersed pioneer of lowland and premontane forest. *Palicourea guianensis* Aubl. (Rubiaceae) is a clonal shrub or treelet (to about 3.5 m) of lowland and premontane moist forest and is often found at the edge of forest clearings. *Poulsenia armata* (Miq.) Standl. (Moraceae) is a shade-tolerant subcanopy species found in relatively fertile soils and riparian habitats in moist lowland and premontane forests. *Symphonia globulifera* L. f. (Clusiaceae) is a shade-tolerant subcanopy to canopy tree (≥30 meters) found along rivers and in upland forests in tropical Africa and the Neotropics including West Indies (Dick et al. 2003; Dick and Heuertz 2008). *Trema micrantha* L. (Ulmaceae) is a small (2–20 m), bird-dispersed pioneer tree found along forest edges; it is a species complex that contains geographically overlapping clades (Yesson et al. 2004). This study is focused on a clade identified as *Trema* sp. 11 by Yesson et al. (2004).

The study species are broadly representative of the Amazon tree flora: ten of the study species are animal-dispersed and two are wind-dispersed species, thus generally matching the proportions of seed dispersal syndromes found in tropical forests. Moreover, the species span the heliophile-shade tolerance spectrum and the understory, canopy and emergent stature continuum, two key axes of differentiation among lowland rain forest trees (Poorter et al. 2006) (Table 1).

### Laboratory methods

Each DNA aliquot was assigned an identification number (Lab ID) linked to associated GPS coordinates, herbarium vouchers, or forest inventory tag numbers (Table S1). Molecular analyses were based on ca. 2400 bp of coding and non-coding chloroplast DNA (*rbc*L, *trn*H-*psb*A, *psb*B-*psb*F) and ca. 700 bp of the ITS region. Forward and reverse DNA strands were sequenced for each genomic region. Laboratory methods have described in detail elsewhere (Dick et al. 2007; Dick and Heuertz 2008). Haplotype networks were constructed using statistical parsimony (Clement et al. 2000). Homopolymer regions (cpDNA simple sequence repeats) were excluded from analyses due to DNA sequence alignment uncertainty and a high likelihood of homoplasy.

### Coalescent analysis

We performed a Bayesian uncorrelated molecular clock analysis (Drummond et al. 2006) using the program BEAST (Drummond and Rambaut 2007) to estimate the time of most recent common ancestor (tMRCA) of the phylogeographic lineages for each species. The analysis was performed on the ITS dataset, for which many fossil-calibrated substitution rates are available (reviewed in Dick et al. (2003) and Kay et al. (2006)). Rate estimates for the ITS region in woody plants range from 0.5 to 3.3 × 10^−9^ substitutions per site per year (s/s/yr) (eight studies) with a mean of 1.64 × 10^−9^. We excluded rates for herbaceous plants, because of their twofold higher rate of nucleotide substitution (Smith and Donoghue 2008), and rates calibrated by vicariance events because, by excluding potential recent dispersal scenarios, these estimates are biased toward slow rates and older age estimates. We used the mean substitution rate, and the slowest and fastest rates for the lower and upper bounds of the standard deviation of the mean. The analyses were run using the HKY substitution model, a lognormal branch length distribution, and constant population size for 10^7^ generations, with data sampled every 10^3^ generations. Effective sample sizes (ESS) of coalescent genealogies exceeded 1000 for all tMCRA estimates. XML input files and MCMC log files are available upon request.

## Results

**Figure 2:**
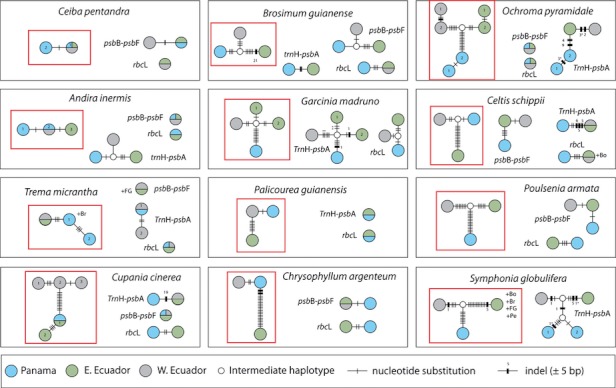
Haplotype networks for nuclear ITS and chloroplast DNA sequence data. Circles represent haplotypes, colors are geographic locations; hatch marks represent mutational steps. ITS haplotypes are bounded by red rectangles. Additional geographic representation of some haplotypes is indicated by country abbreviations: Bolivia (Bo), Brazil (Br), French Guiana (FG), and Peru (Pe).

Despite the slow mutation rates of the genetic markers, which are often used in phylogenetic studies, a majority of the species exhibited strong phylogeographic structure as indicated in the haplotype networks (Fig. 2). Mean tMRCA estimates ranged from 0.22 (±0.006) to 9.88 (±0.021) Ma (Table 1). Nine of the 12 species had a Neogene tMRCA; seven species were of Miocene age (Table 1). Our results indicate that geographic lineages in most of the studied species (nine of 12 species) originated during the Neogene and that some of these populations passed through temperatures that were warmer than the present day.

## Discussion

**Figure 3:**
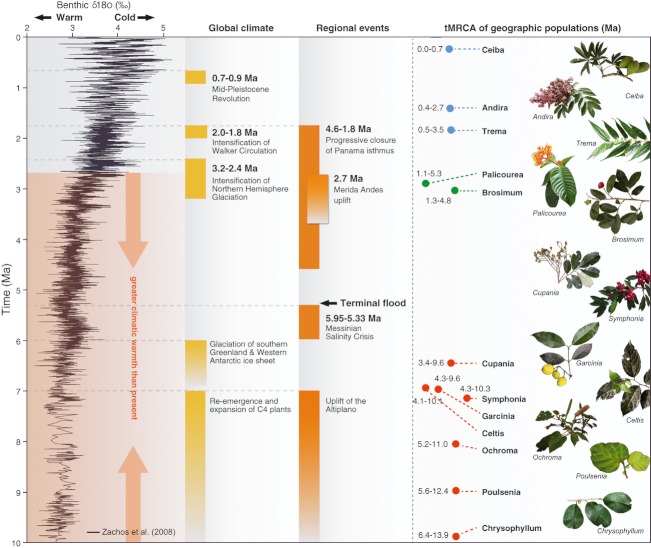
tMRCA of geographic lineages and relevant corresponding surface air temperatures (Zachos et al. 2008) and climatic and geological events. Mean tMRCA estimates are shown in colored circles (Pleistocene blue, Pliocene green, Miocene red) accompanied by upper and lower 95% HPD intervals. Large vertical arrows indicate period in which average surface air temperatures were warmer than present. Tree photographs were provided by Center for Tropical Forest Sciences.

Although Pleistocene-age species have been documented within at least one large Amazon tree genus *Inga* (Richardson et al. 2001), our study suggests that a cross-section of Amazon tree diversity may be much older. The results demonstrate the persistence of geographically structured populations of rain forest trees through several recent geological epochs and through major geological and climatic changes (Fig. 3). The nine Neogene-aged species have witnessed the Merida uplift, and the progressive closing of the Isthmus of Panama (ca. 3.1 Ma) (Coates and Obando 1996). The significant global climatic changes (Zachos et al. 2008; Willis et al. 2010) include (1) late-Miocene and early-Pliocene warm periods; (2) the intensification of Northern Hemisphere Glaciation (3.2–2.5 Ma); (3) the Mid-Pleistocene Revolution (ca. 1–0.8 Ma), when mean tropical temperatures decreased, but variability increased on both orbital and millennial time-scales; and (4) the intensification of the Walker Circulation (ca. 1.9 Ma), which increased the seasonality of the hydrological cycle and the sensitivity of the tropics to precessional cycles (Maslin et al. 2005) (Fig. 3). Many of these older species occurred in regions in which no upper elevation refuges were available during periods of global warmth, suggesting that these species, and rain forest tree species more broadly, have higher thermal tolerance than niche models and some other studies have suggested (Thomas et al. 2004; Colwell et al. 2008). However, we cannot discount the possibly that some higher temperature tolerance may have been lost over time. This hypothesis should be a priority future research focus.

Molecular clock analyses are subject to possible errors caused by rate variation across lineages, incorrect phylogenetic placement of fossils for time calibration, and incomplete sorting of ancestral polymorphisms. Our study accounts for error associated with rate variation and genealogical stochasticity by incorporating the full range of relevant substitution rates into the Bayesian coalescent analysis. The application of even the fastest substitution rate in our study places the origin of 7 of the 12 species in the Neogene (Table 1). It is possible that some divergent haplotypes could be derived from introgression from related species. However, four of the older species are from genera (*Symphonia*, *Celtis*, *Poulsenia*, *Ochroma*) that each contains only one tree species in Neotropical forests, and thus these species are unable to hybridize or share ancestral variation with congeneric tree species. In fact, because we did not sample across the full geographic range of these species, some basal phylogeographic lineages may be unsampled. Our results, therefore, provide a conservative estimate of the species ages.

The fossil record for our best-studied and most widespread species, *Symphonia globulifera*, corroborates the times scales presented here (Dick et al. 2003; Dick and Heuertz 2008). The earliest *Symphonia* pollen occurs in mid-Eocene deposits (ca. 45 Ma) in Nigeria, and *S. globulifera* pollen appears in mid-Miocene sediments in Venezuela and Brazil (ca. 15 Ma), and later in mid-Pliocene sediments in Mexico and Southeast Costa Rica. The mean tMRCA for *S*. *globulifera* reported here (ca. 7 Ma) is substantially younger than its oldest Neotropical fossils (ca. 15 Ma) (Dick et al. 2003).

There are at least two demographic survival scenarios for these old species. Populations may have persisted in lowland forests suggesting that the lineages have survived past high air temperature levels. Alternatively, the species may have tracked higher elevation areas with temperatures matching their physiological optima. Populations located in the western Amazon could find refuge on the slopes of the Andes, although these mountains were smaller in the Neogene (Hoorn et al. 2010). However, many of the species have disjunct populations in the West Indies and the Atlantic forests of Brazil (Table 1), where there were no nearby upper elevation refuges. Moreover, fossil evidence suggests forest stability in the core Amazon during the Neogene (Silva et al. 2010) rather than forest area contractions or the formation of lowland refugia.

One possible difference between species survival through past warm climates and those projected for the 21st century is the rate of temperature increase (Thomas et al. 2004; Botkin et al. 2007). Past climatic changes are thought to have included rapid warming events (Zachos et al. 2008) that, coupled with long tree generation times, suggest that acclimatization or standing adaptive variation was more important for climate response than major evolutionary innovation. Thus, decreases in effective population size and the loss of phylogeographic structure, such as that resulting from forest removal or fragmentation, are the causes for concern. The likely human-induced loss of genetic variation represents a novel difference when comparing 21st century Amazon rain forest with previous epochs, and may well impact the adaptive response of tree species to temperature increases and other environmental changes.

The phylogeographic data presented here relate to species persistence and not to other forest attributes. Although experimental evidence suggests that tropical trees may physiologically tolerate a warmer climate than today (Krause et al. 2010), some experimental and eddy-covariance flux data demonstrate that tropical trees may be closed to, but have not exceeded, their temperature optima for carbon acquisition (Doughty and Goulden 2008; Lloyd and Farquhar 2008; Lewis et al. 2009). All else being equal, higher temperatures in the future will increase carbon losses from respiration, while midday photosynthesis will be reduced, lowering productivity and tree biomass (Lloyd and Farquhar 2008; Lewis et al. 2009). These impacts may then be reinforced by drought impacts (Lewis et al. 2011), or ameliorated by higher atmospheric carbon dioxide levels (Lloyd and Farquhar 2008). Depending upon the strength of the fertilization effect of atmospheric carbon dioxide on tree growth, warming may reverse the current net carbon uptake by intact Amazon forests (Phillips et al. 2008), even if most species persist (e.g., see Galbraith et al. 2010; Rammig et al. 2010).

Climate projections for Amazonia include not only higher air temperatures, but some models project an increasing frequency and intensity of drought events (Malhi et al. 2008), such as those seen in 2005 and 2010 (Lewis et al. 2011). This may lead to lower biomass forest, replacement by novel forest types, or non-forest vegetation via forest dieback (Galbraith et al. 2010; Rammig et al. 2010). Yet, intriguingly, large-scale dieback during high temperature or drought events is not supported by paleoecological evidence (e.g., Mayle and Power 2008). The differences between model projections and paleoecological studies may be due to the non-analogue conditions of 21st century Amazonia, but they could also stem from a lack of a paleoecological perspective within the biosphere modeling community. Our study cautiously suggests that many Amazon tree species can likely tolerate increasing air temperatures to some degree. This implies that drought events, direct human impacts (forest clearance, forest fragmentation, fires, and loss of seed dispersing animals), and their interactions may be more important immediate threats to the integrity of Amazon rain forests and therefore should remain a focus of conservation policy.

## Supporting Information

**Table S1.** Accession and voucher information for sequenced individuals of the 12 study species. Samples were collected from localities within Ecuador (EC), Panama (PA), Brazil (BR), Bolivia (Bo), and French Guiana (FG). Tag number references trees collected in permanent forest inventory plots. The ID refers to the catalog number of DNA archived at the University of Michigan.
