# Supplementary material for: Neogene origins and implied warmth tolerance of Amazon tree species
Source: Ecol Evol. 2013 Jan 10;3(1):162–9. doi: 10.1002/ece3.441 (PMC3568851; doi:10.1002/ece3.441)
Supplement: Supplementary file 1 [file ece30003-0162-SD1.docx]

| Locality | ID | Plot # | ITS | *trn*H-*psb*A | *psb*B-*psb*F | *rbc*L |  |
| --- | --- | --- | --- | --- | --- | --- | --- |
| *Andira inermis* | | | | | | |  |
| Yasuní, EC | 1 | 85784 | AY635469 | JX997366 | JX987644 | JX987565 |  |
| Yasuní, EC | 2 | 123029 | AY635470 | JX997365 | JX987645 |  |  |
| Yasuní, EC | 3 | 123190 | AY635471 |  |  |  |  |
| Yasuní, EC | 4 | 152644 | AY635472 |  |  |  |  |
| Yasuní, EC | 5 | 125744 | AY635473 |  |  |  |  |
| Esmeraldas, EC | 868 |  | AY635474 | JX997361 | JX987640 |  |  |
| Esmeraldas, EC | 870 |  | AY635475 |  |  |  |  |
| Esmeraldas, EC | 872 |  | AY635476 | JX997362 | JX987641 |  |  |
| BCI, PA | 304 | 271538 | AY635477 | JX997364 | JX987642 | JX987567 |  |
| BCI, PA | 305 | 218784 | AY635478 |  |  |  |  |
| BCI, PA | 979 | 462999 | AY635479 | JX997363 | JX987643 | JX987566 |  |
| BCI, PA | 980 |  | AY635480 |  |  |  |  |
| *Brosimum guianense* | | | | | | |  |
| Yasuní, EC | 11 | 213588 | AY635489 | JX997334 | JX987656 |  |  |
| Yasuní, EC | 12 | 224342 | AY635492 |  |  |  |  |
| Yasuní, EC | 13 | 244658 | AY635490 |  |  |  |  |
| Yasuní, EC | 14 | 244118 | AY635491 |  | JX987657 | JX987570 |  |
| Esmeraldas, EC | 841 |  | AY635481 | JX997336 |  |  |  |
| Esmeraldas, EC | 845 |  | AY635482 |  |  |  |  |
| Esmeraldas, EC | 863 |  | AY635483 | JX997335 | JX987658 |  |  |
| Esmeraldas, EC | 882 |  |  | JX997337 |  |  |  |
| Kourou, FG | 452 | PB011 | AY635484 |  |  | JX987569 |  |
| Manaus, BR | 8 | 1202.3064 | AY635485 |  |  |  |  |
| Manaus, BR | 9 | 41.068 | AY635486 |  |  |  |  |
| Ft. Sherman, PA | 540 | 28724 | AY635487 |  | JX987659 | JX987568 |  |
| Ft. Sherman, PA | 542 | 28699 | AY635488 |  | JX987660 |  |  |
| *Ceiba pentandra* | | | | | | |  |
| Yasuní, EC | 65 | 111765 | AY635500 |  | DQ284793 |  |  |
| Yasuní, EC | 66 |  | AY635501 |  | DQ284794 |  |  |
| Yasuní, EC | 67 |  | JX512554 |  | DQ284795 |  |  |
| Yasuní, EC | 1021 |  | DQ284833 |  | DQ284797 | JX987572 |  |
| Esmeraldas, EC | 63 |  | AY635495 |  |  |  |  |
| Esmeraldas, EC | 298 |  | AY635494 |  | AY642681 |  |  |
| Esmeraldas, EC | 867 |  | DQ284827 |  | DQ284788 |  |  |
| Esmeraldas, EC | 871 |  | DQ284828 |  | DQ284789 |  |  |
| Esmeraldas, EC | 875 |  | DQ284829 |  | DQ284790 |  |  |
| Esmeraldas, EC | 883 |  | DQ284830 |  | DQ284791 | JX987571 |  |
| Los Santos, PA | 518 |  | AY635497 |  | AY642675 |  |  |
| Los Santos, PA | 519 |  | AY635498 |  | AY642682 |  |  |
| Los Santos, PA | 520 |  | DQ284825 |  | DQ284786 |  |  |
| *Celtis schippii* | | | | | | |  |
| Yasuní, EC | 70 | 194444 | AY635509 | JX997338 | JX987619 | JX987576 |  |
| Yasuní, EC | 72 |  |  |  | JX987615 | JX987574 |  |
| Yasuní, EC | 74 | 244744 | AY635510 |  |  |  |  |
| Yasuní, EC | 1022 |  | JX987529 |  |  |  |  |
| Esmeraldas, EC | 77 |  | AY635505 | JX997339 | JX987617 | JX987575 |  |
| Esmeraldas, EC | 78 |  | AY635506 |  | JX987620 |  |  |
| Esmeraldas, EC | 963 |  | JX987527 | JX997340 |  |  |  |
| BCI, PA | 75 | 118848 | AY635507 | JX997341 | JX987616 | JX987577 |  |
| BCI, PA | 76 | 107836 | AY635508 | JX997342 | JX987618 | JX987578 |  |
| Pipeline Rd., PA | 1333 |  | JX987530 |  |  |  |  |
| Pipeline Rd., PA | 1345 |  | JX987531 |  |  |  |  |
| Valle del Jacta, BO | 945 |  | JX987528 | JX997343 |  | JX987573 |  |
| *Chrysophyllum argenteum* | | | | | | |  |
| Pipeline Rd, PA | 1303 |  | JX987535 |  |  |  |  |
| Pipeline Rd, PA | 1316 |  | JX987536 |  |  |  |  |
| Pipeline Rd, PA | 1079 |  | JX987537 |  |  |  |  |
| BCI, PA | 79 | 113113 | AY635513 |  | JX987665 | JX987580 |  |
| BCI, PA | 80 | 109100 | AY635514 |  | JX987662 | JX987581 |  |
| Esmeraldas, EC | 81 |  | AY635511 |  | JX987666 |  |  |
| Esmeraldas, EC | 82 |  | AY635512 |  | JX987663 |  |  |
| Esmeraldas, EC | 835 |  | JX987538 |  |  |  |  |
| Yasuní, EC  Yasuní, EC | 84  85 | 223821 | JX987532  AY635515 |  | JX987664 |  |  |
| Yasuní, EC | 86 |  | JX987534 |  |  |  |  |
| Yasuní, EC | 87 | 243743 | AY635516 |  | JX987661 |  |  |
| Yasuní, EC | 1030 | 11253 | JX987533 |  |  | JX987579 |  |
| *Cupania cinerea* | | | | | | |  |
| Pipeline Rd., PA | 1375 |  | JX987540 |  |  |  |  |
| BCI, PA | 96 | 94840 | AY635523 | JX997348 | JX987635 | JX987584 |  |
| BCI, PA | 1007 | 418566 | JX987539 |  | JX987636 | JX987585 |  |
| Esmeraldas, EC | 97 |  | AY635522 |  | JX987639 |  |  |
| Esmeraldas, EC | 849 |  | AY635517 |  |  |  |  |
| Esmeraldas, EC | 850 |  | AY635518 | JX997350 |  |  |  |
| Esmeraldas, EC | 853 |  | AY635519 |  |  |  |  |
| Esmeraldas, EC | 864 |  | AY635520 | JX997349 |  |  |  |
| Esmeraldas, EC | 877 |  | AY635521 |  |  |  |  |
| Yasuní, EC | 98 | 152683 | AY635525 |  | JX987637 | JX987582 |  |
| Yasuní, EC | 99 | 152519 | AY635526 | JX997347 |  |  |  |
| Yasuní, EC | 100 | 191938 | AY635524 |  | JX987638 | JX987583 |  |
| *Garcinia madruno* | | | | | | |  |
| Yasuní, EC | 113 | 183208 | AY635535 |  | JX987648 |  |  |
| Yasuní, EC | 114 | 95579 | AY635536 |  |  |  |  |
| Yasuní, EC | 115 | 105191 | AY635537 | JX997352 |  |  |  |
| Yasuní, EC | 116 | 164481 | AY635538 | JX997351 |  | JX987587 |  |
| Esmeraldas, EC | 111 |  | AY635527 | JX997356 | JX987647 | JX987586 |  |
| Esmeraldas, EC | 112 |  | AY635528 | JX997355 |  |  |  |
| Esmeraldas, EC | 961 |  | AY635529 |  |  |  |  |
| Esmeraldas, EC | 962 |  | AY635530 |  |  |  |  |
| Esmeraldas, EC | 964 |  |  |  |  |  |  |
| Esmeraldas, EC | 977 |  | AY635531 |  |  |  |  |
| Esmeraldas, EC | 978 |  | JX987543 |  |  |  |  |
| BCI, PA | 110 | 88184 | AY635532 |  | JX987646 | JX987588 |  |
| BCI, PA | 301 | 228914 | AY635533 | JX997353 |  | JX987589 |  |
| BCI, PA | 983 | 144027 | AY635534 | JX997354 |  |  |  |
| BCI, PA | 981 | 133160 | JX987541 |  |  | JX987590 |  |
| BCI, PA | 982 | 144012 | JX987542 |  |  |  |  |
| *Ochroma pyramidale* | | | | | | |  |
| Yasuní, EC | 183 |  | AY635548 | JX997368 |  |  |  |
| Yasuní, EC | 184 |  | AY635549 |  | JX987607 |  |  |
| Yasuní, EC | 185 |  | AY635550 | JX997369 |  |  |  |
| Yasuní, EC | 186 |  | AY635551 |  | JX987608 | JX987591 |  |
| Esmeraldas, EC | 180 |  | AY635539 |  | JX987614 |  |  |
| Esmeraldas, EC | 181 |  | AY635540 |  | JX987613 |  |  |
| Esmeraldas, EC | 964 |  | AY635541 | JX997370 |  | JX987594 |  |
| Esmeraldas, EC | 965 |  | AY635542 |  |  |  |  |
| Soberanía, PA | 182 |  | AY635543 |  | JX987611 |  |  |
| Los Santos, PA | 528 |  | AY635544 | JX997371 | JX987603 | JX987595 |  |
| Los Santos, PA | 530 |  | AY635545 |  | JX987612 |  |  |
| BCI, PA | 545 |  | AY635546 | JX997372 | JX987610 |  |  |
| BCI, PA | 546 |  | AY635547 |  | JX987609 |  |  |
| BCI, PA | 1402 |  | JX987548 |  | JX987602 |  |  |
| BCI, PA | 1403 |  | JX987550 |  | JX987606 |  |  |
| BCI, PA | 1404 |  | JX987549 |  | JX987605 |  |  |
| Pipeline Rd, PA | 1405 |  | JX987547 |  |  |  |  |
| Pipeline Rd, PA | 1406 |  | JX987546 |  |  |  |  |
| Pipeline Rd, PA | 1407 |  | JX987545 |  | JX987604 |  |  |
| Valle del Jacta, BO | 942 |  | JX987544 | JX997367 |  |  |  |
| *Palicourea guianensis* | | | | | | |  |
| Yasuní, EC | 193 | 244320 | AY635556 |  |  |  |  |
| Yasuní, EC | 194 |  | AY635557 |  |  |  |  |
| Yasuní, EC | 195 | 244741 | AY635558 | JX997345 |  |  |  |
| Yasuní, EC | 196 | 244406 | AY635559 | JX997344 |  | JX987597 |  |
| Yasuní, EC | 197 | 233817 | AY635560 |  |  |  |  |
| Esmeraldas, EC | 191 |  | AY635552 |  |  |  |  |
| Esmeraldas, EC | 192 |  | AY635553 |  |  |  |  |
| BCI, PA | 187 | 421169.1 | AY635554 | JX997346 |  | JX987596 |  |
| BCI, PA | 188 | 421169.2 | AY635555 |  |  |  |  |
| *Poulsenia armata* | | | | | | |  |
| Yasuní, EC | 202 | 244273 | AY635564 |  | JX987627 |  |  |
| Yasuní, EC | 203 | 243640 | AY635565 |  | JX987628 |  |  |
| Esmeraldas, EC | 200 |  | AY635561 |  | JX987629 |  |  |
| BCI, PA | 198 | 118771 | AY635562 |  | JX987623 | JX987592 |  |
| BCI, PA | 199 | 107545 | AY635563 |  | JX987621 |  |  |
| BCI, PA | 1393 |  | JX987551 |  | JX987630 |  |  |
| BCI, PA | 1394 |  | JX987552 |  | JX987631 |  |  |
| BCI, PA | 1395 |  | JX987553 |  | JX987632 |  |  |
| BCI, PA | 1396 |  | JX987554 |  | JX987633 |  |  |
| BCI, PA | 1397 |  | JX987555 |  | JX987634 |  |  |
| Pipeline Rd., PA | 1390 |  | JX987556 |  | JX987624 |  |  |
| Pipeline Rd., PA | 1391 |  | JX987557 |  | JX987625 |  |  |
| Pipeline Rd., PA | 1392 |  | JX987558 |  | JX987626 |  |  |
| Valle del Jacta, BO | 944 |  |  |  | JX987622 |  |  |
| *Symphonia globulifera* | | | | | | |  |
| BCI, PA | 252 | 218310 | AY633884 |  |  |  |  |
| BCI, PA | 253 | 2457 | AF479784 | EU841724 |  |  |  |
| Jatun Sacha, EC | 257 | 2457 | AY633885 | EU841869 |  |  |  |
| Manaus, Br | 258 |  | AY633886 |  |  |  |  |
| Esmeraldas, EC | 260 |  | AY633887 | EU841763 |  |  |  |
| Esmeraldas, EC | 261 |  | AY633888 | EU841764 |  |  |  |
| Yasuní, EC | 263 | 104514 | AY633889 | EU841870 |  |  |  |
| Paracou, FG | 394 | PB275 | AY633890 |  |  |  |  |
| Paracou, FG | 404 | 1610417 |  |  |  |  |  |
| Paracou, FG | 406 | 1610245 | AF479786 | EU841775 |  |  |  |
| Paracou, FG | 407 | 161025 | AY633891 | EU841776 |  |  |  |
| Paracou, FG | 410 | 16142677 |  |  |  |  |  |
| BCI, PA | 497 | 4733 | AY633892 | EU841725 |  |  |  |
| BCI, PA | 500 | 7831 | AY633893 |  |  |  |  |
| BCI, PA | 560 | 54076 | AY633894 | EU841727 |  |  |  |
| BCI, PA | 563 | 18273 |  |  |  |  |  |
| BCI, PA | 570 | 307491 |  | EU841728 |  |  |  |
| Manaus, BR | 641 | 2303.3616 | AY633895 | EU841818 |  |  |  |
| Manaus, BR | 642 | 3304.5251 | AY633896 | EU841819 |  |  |  |
| Manaus, BR | 643 | Km41.346 | AY633897 | EU841820 |  |  |  |
| Yasuní, EC | 647 | 84144 | AY633898 | EU841871 |  |  |  |
| Yasuní, EC | 651 | 32396 | AY633899 | EU841872 |  |  |  |
| Esmeraldas, EC | 657 |  | AY633900 | EU841765 |  |  |  |
| Esmeraldas, EC | 658 |  | AF479785 | EU841757 |  |  |  |
| Esmeraldas, EC | 660 |  | AY633901 |  |  |  |  |
| Esmeraldas, EC | 661 |  | AY633902 |  |  |  |  |
| Ft. Sherman, PA | 926 |  | AY633903 |  |  |  |  |
| Valle del Jacta, BO | 940 |  | AY633904 | EU841729 |  |  |  |
| Valle del Jacta, BO | 941 |  | AY633905 | EU841730 |  |  |  |
| BCI, PA | 984 | 289410 |  |  |  |  |  |
| BCI, PA | 985 | 292095 | AY633907 |  |  |  |  |
| BCI, PA | 986 | 307491 | AY633908 |  |  |  |  |
| BCI, PA | 987 | 307515 | AY633909 |  |  |  |  |
| BCI, PA | 989 | 307518 | AY633910 |  |  |  |  |
| BCI, PA | 990 | 307519 | AY633911 |  |  |  |  |
| BCI, PA | 991 | 310316 | AY633912 |  |  |  |  |
| BCI, PA | 992 | 308909 | AY633913 |  |  |  |  |
| BCI, PA | 993 | 310526 | AY633914 |  |  |  |  |
| BCI, PA | 994 | 310606 | AY633915 |  |  |  |  |
| Yasuní, EC | 995 | 310622 | AY633916 |  |  |  |  |
| Yasuní, EC | 996 | 234163 | AY633917 |  |  |  |  |
| Yasuní, EC | 997 | 200474 | AY633918 |  |  |  |  |
| Yasuní, EC | 998 | 71744 | AY633919 |  |  |  |  |
| Yasuní, EC | 999 | 71227 | AY633920 |  |  |  |  |
| Yasuní, EC | 1000 | 246118 | AY633921 |  |  |  |  |
| Yasuní, EC | 1001 | 84144 | AY633922 |  |  |  |  |
| Yasuní, EC | 1003 | 121550 | AY633923 |  |  |  |  |
| Yasuní, EC | 1004 | 220737 | AY633924 |  |  |  |  |
| Yasuní, EC | 1005 | 10175 | AY633925 |  |  |  |  |
| Yasuní, EC | 1006 | 244949 |  |  |  |  |  |
| *Trema micrantha* | | | | | | |  |
| Yasuní, EC | 278 |  | AY635570 |  | JX987653 |  |  |
| Yasuní, EC | 279 |  | AY635571 |  | JX987651 |  |  |
| Esmeraldas, EC | 276 |  |  |  |  |  |  |
| Esmeraldas, EC | 277 |  |  |  | JX987654 |  |  |
| Yasuní, EC | 281 |  | JX987559 |  |  | JX987600 |  |
| Esmeraldas, EC | 973 |  | JX987560 | JX997359 |  | JX987601 |  |
| Esmeraldas, EC | 974 |  | JX987561 | JX997358 |  |  |  |
| Esmeraldas, EC | 972 |  | JX987564 | JX997357 |  | JX987599 |  |
| Kourou, FG | 363 |  | AY635566 |  | JX987655 |  |  |
| Kourou, FG | 364 |  | AY635567 |  |  |  |  |
| Kourou, FG | 365 |  | AY635568 |  | JX987652 |  |  |
| BCI, PA | 535 |  |  | JX997360 | JX987649 | JX987598 |  |
| BCI, PA | 536 |  | AY635569 |  | JX987650 |  |  |
| BCI, PA | 537 |  | JX987562 |  |  |  |  |
| BCI, PA | 538 |  | JX987563 |  |  |  |  |

SI Table 1. Genbank accession and voucher information.
